# Wnt signalling mediates miR-133a nuclear re-localization for the transcriptional control of *Dnmt3b* in cardiac cells

**DOI:** 10.1038/s41598-019-45818-4

**Published:** 2019-06-27

**Authors:** Vittoria Di Mauro, Silvia Crasto, Federico Simone Colombo, Elisa Di Pasquale, Daniele Catalucci

**Affiliations:** 1 University of Milan Bicocca, Piazza dell’Ateneo Nuovo 1, 20126 Milan, Italy; 2 CNR-IRGB UOS Milan, Via Fantoli 15/16, 20138 Milan, Italy; 3 Humanitas Clinical and Research Center, via Alessandro Manzoni 113, 20089 Rozzano, Milan Italy

**Keywords:** Cardiovascular biology, miRNAs

## Abstract

MiR-133a is a muscle-enriched miRNA, which plays a key role for proper skeletal and cardiac muscle function via regulation of transduction cascades, including the Wnt signalling. MiR-133a modulates its targets via canonical mRNA repression, a process that has been largely demonstrated to occur within the cytoplasm. However, recent evidence has shown that miRNAs play additional roles in other sub-cellular compartments, such as nuclei. Here, we show that miR-133a translocates to the nucleus of cardiac cells following inactivation of the canonical Wnt pathway. The nuclear miR-133a/AGO2 complex binds to a complementary miR-133a target site within the promoter of the de novo DNA methyltransferase 3B (Dnmt3b) gene, leading to its transcriptional repression, which is mediated by DNMT3B itself. Altogether, these data show an unconventional role of miR-133a that upon its relocalization to the nucleus is responsible for epigenetic repression of its target gene Dnmt3b via a DNMT3B self-regulatory negative feedback loop.

## Introduction

The multifactorial nature of the cardiac system tightly relies on the dynamic and regulated interplay between signalling pathways and the modulation of associated effector molecules. In this contest, of relevant importance is the canonical Wnt signalling, which has been demonstrated to tightly regulate multiple aspects of both cardiac development and maintenance of adult heart homeostasis^1^. Specifically, without Wnt protein stimulation (Wnt *off-state*), the main effector, β-catenin, is anchored to the cell membrane by a destruction complex comprised of APC, GSK3β, and Axin, preventing it from performing its action. After an initial step of phosphorylation by GSK-3β and CK1α, β-catenin is marked for proteasomal degradation. On the contrary, activation of the cascade (Wnt *on-state*) stabilizes β-catenin, which in turn translocates to the nucleus, where it binds to members of the lymphoid enhancement factor/T-cell factor (LEF/TCF) family of transcription factors. β-catenin binding converts LEF/TCF factors from repressors to activators, thereby initiating cell-specific gene transcription patterns^2^. Based on the importance of canonical Wnt signalling, it is well accepted that even slight perturbations in the regulation of this cascade can result in the onset of pathological disorders that might occur during heart development or myocardial homeostasis in the adult heart^3^. In line with this, emerging evidence has indicated the importance of the interaction between the Wnt β-catenin signalling pathway and microRNA (miRNA)-mediated gene regulation in the cardiovascular field^2^. The miRNA family represents the so far most well studied group of non-coding RNAs (ncRNAs), belonging to the 20–25 nucleotide small ncRNA class, whose function has been extensively studied in the cytosolic compartment, where miRNAs selectively induce post-transcriptional gene silencing of target genes through guidance of the RNA induced silencing complex (RISC) to translationally repress, or even degrade, complementary sequence-matched messenger RNA (mRNA) targets^4–6^. However, recent gene expression and deep sequencing analyses performed on different subcellular compartments have revealed an unexpected presence of mature miRNAs also within other sub-cellular organelles, such as the endoplasmic reticulum (ER)^7^, mitochondria^8^, and nuclei^9^, thus envisioning additional and more widespread functions. In fact, accumulating evidence has shown that mature miRNAs together with components of the RISC, can translocate to the nucleus and effectively modulate gene as well as ncRNA expression directly at the genomic level either through inhibition or activation^10–13^. However, notwithstanding this new role of miRNAs in orchestrating gene activities was reported in cancer or hematopoiesis^14^, it has remained unknown whether miRNAs show similar roles in other cellular contexts, including cardiac cells. In particular, no evidence for differential sub-cellular relocalization and repression of genomic targets has so far been provided for the cardiac-enriched miR-133a.

Here, we identified an unconventional role of miR-133a, demonstrating the translocation of its mature form to the nucleus following inhibition of the Wnt signalling pathway. Through binding to a specific miR-133a target site within the promoter region of the *de novo* DNA methyltransferase 3B gene (*Dnmt3b*), nuclear AGO2-bound miR-133a initiates an epigenetic control of *Dnmt3b* gene transcription repression, which enrols DNMT3B for a self-regulatory circuit.

## Results

### Inhibition of the canonical Wnt signalling pathway induces nuclear enrichment of miR-133a

**Figure 1:**
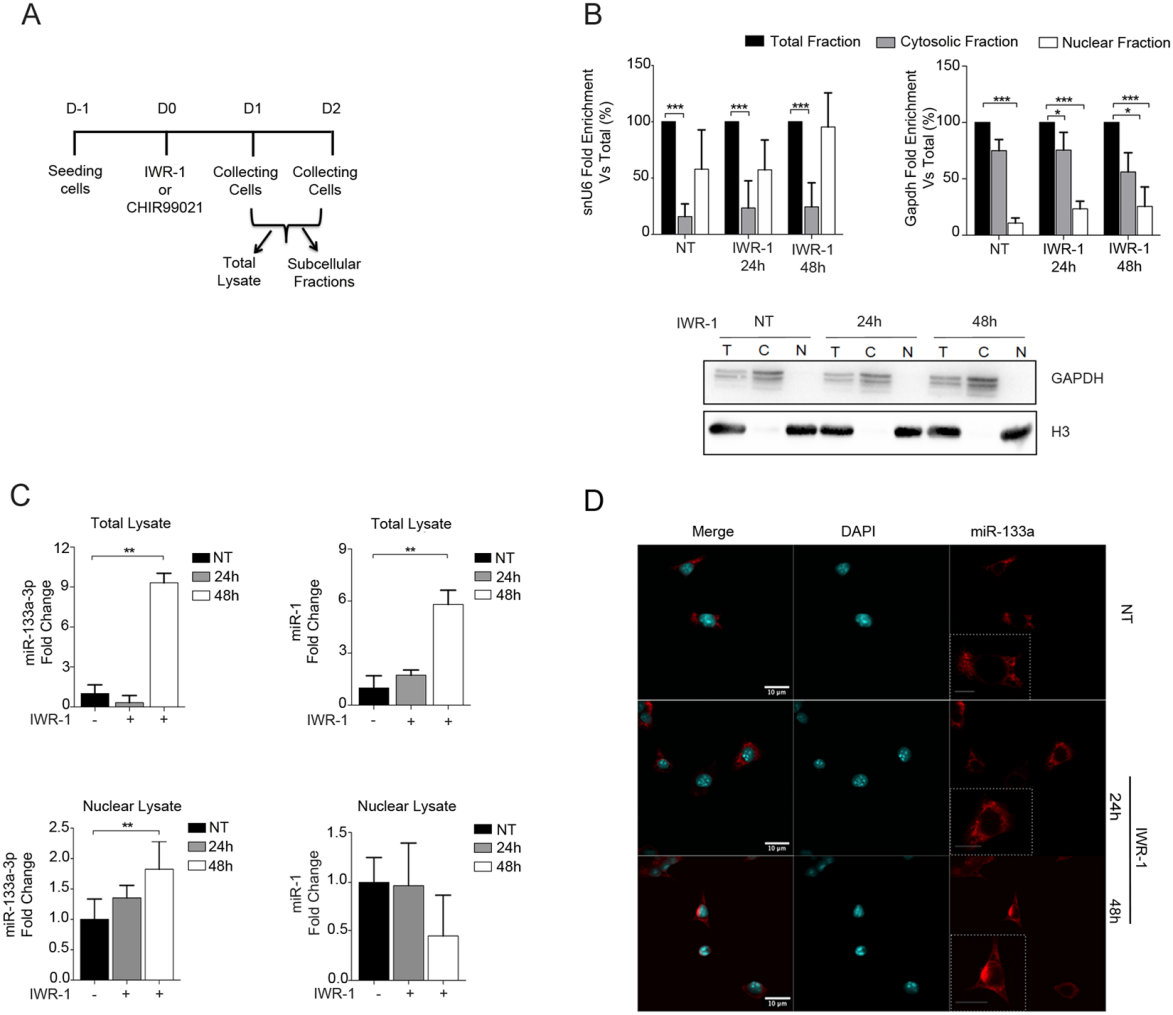
Sub-cellular distribution of mature miRNAs after chemical inhibition of the Wnt/β-catenin signalling pathway in HL-1 cells. (**A**) Schematic representation of cellular treatment and sub-cellular fractionation. (**B**) Control of fractionation purity by qRT-PCR (*snU6* and *Gapdh*) and Western blot (cropped images of parts of the same gel) analysis (GAPDH and H3). (**C**) qRT-PCR for miR-133a and miR-1 levels in total cell lysates and purified nuclear fractions. (**D**) Representative FISH image of HL-1 cells co-stained for miR-133a (red) and DAPI (blue) for nuclei visualization (n = 5); *p < 0.05; **p < 0.01.

A plethora of evidence has indicated that active transcription of miR-1 and miR-133a, known as myomiRs, is associated with proper homeostasis of the cardiac system^15^. Among the triggering transduction cascades affecting these myomiRs, the Wnt β-catenin signalling pathway has recently found increasing relevance^1,16^. To assess whether the subcellular distribution of miR-133a and miR-1 might be affected by the activation status of the Wnt transduction cascade, we determined myomiR levels in cytosolic and nuclear fractions of the HL-1 murine cardiac cell line following treatment with either Wnt inhibitor (IWR-1) or activator (CHIR99021)^17,18^. The efficiency of IWR-1 or CHIR99021 treatment was confirmed by determination of mRNA levels of selected downstream targets of the canonical Wnt signalling cascade, such as *Pparδ*, *Gata6*, *Vegfα*, and *Ccdn1* (Fig. S1). The purity of cytosolic and nuclear fractions isolated 24 and 48 hours post IWR-1 treatment (Fig. 1A) was verified by evaluation of cytoplasmic (GAPDH) and nuclear markers (U6 snRNA and histones) by qRT-PCR and Western blot analysis, respectively (Fig. 1B). Notably, we found that total levels of both miR-133a and miR-1 increased in the Wnt *off-state* (Fig. 1C). However, despite an overall larger presence in the cytosol for both miRNAs (Fig. S2A), only miR-133a was significantly enriched in the nuclear compartment (Fig. 1C). In order to evaluate whether inactivation of Wnt canonical signaling could affect other muscle-enriched miRNAs, such as miR-206 and miR-208a^19–21^, we also checked the sub-cellular distribution of these additional miRNAs in treated and not treated cells. As shown in supplementary Fig. S3, both miRNAs are present in the cytosolic compartment as well as in the nucleus of HL-1. However, this nuclear detection is not linked to the IWR-1 treatment, thus while revealing a potential nuclear role also for miR-206 and miR-208a, this is not related to the inactivation of Wnt canonical pathway. Conversely, the total and nuclear profile of miR-19b and miR-34a-5p, two other miRNAs known to have a significant interplay with Wnt canonical signalling^22,23^ did not result with any significant effects on nuclear distribution in response of the treatment (Fig. S2B).

The above results were confirmed by fluorescent *in situ* hybridization (FISH) experiments, where an antisense LNA probe for mature miR-133a showed nuclear enrichment following IWR-1 treatment (Fig. 1D), while an antisense LNA probe for mature miR-1 did not show any nuclear enrichment (Fig. S4A). No signals were detected using scramble-LNA control probe (Fig. S4B).

In contrast, treatment of HL-1 cells with CHIR99021^24^, an inhibitor of GSK-3β activating canonical Wnt signalling (Wnt *on-state*), did not induce any alterations in the relative abundance and subcellular localization of mature miR-133a or miR-1 (Fig. S4C,D).

Altogether, these data indicate that the miR-133a level, but not the miR-1 level, is increased in nuclear compartment following inhibition of the Wnt canonical cascade in HL-1 cardiac cells.

### Inhibition of Wnt canonical signalling increases the nuclear presence of AGO2-loaded miR-133a

**Figure 2:**
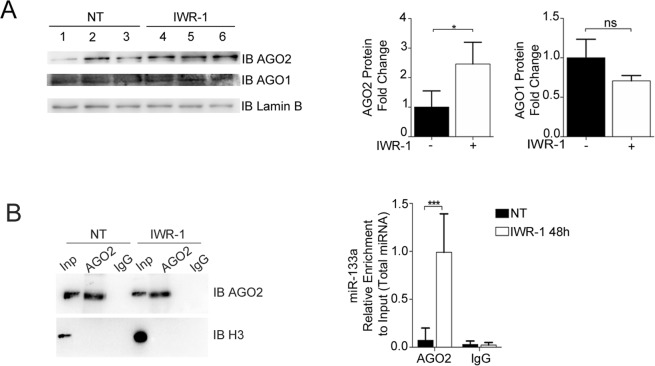
Inhibition of the Wnt/β-catenin signalling pathway induces an increase in AGO2 protein levels into the nucleus of HL-1 cells and its loading with miR-133a. (**A**) Western blot assay and densitometry for protein levels of AGO2 and AGO1 on three biological replicates of nuclear lysates (1-2-3;4-5-6) derived from HL-1 cells treated or not treated (NT) with IWR-1 (48 h). (For Western blot assay cropped image). (**B**) Western blot assay for immunoprecipitated endogenous AGO2 on isolated nuclei of HL-1 cells treated or not with IWR-1 (48 h), and qRT-PCR for miRNAs following endogenous AGO2 immunoprecipitation on isolated nuclei of HL-1 cells stimulated with IWR-1 (n = 3); ***p < 0.01. (For Western blot cropped images of parts of two gels).

To further corroborate the nuclear presence of mature miR-133a as a consequence of Wnt pathway inhibition, we next evaluated whether the nuclear translocation of the miRNA might be mediated by Argonautes (AGOs), a class of evolutionary conserved proteins with a central role in both miRNA biogenesis and function. In fact, although their cytosolic function is widely recognized, AGO1 and AGO2 were recently found in the nucleus of murine and human cells^25–27^. In line with this, we found that both AGO1 and AGO2 were present in nuclear lysates of HL-1 cells independent of whether or not the cells were treated with IWR-1. However, only AGO2 was significantly enriched in the nuclear compartment in the Wnt *off-state* (Fig. 2A) and RNA immunoprecipitation assays showed that the nuclear pool of AGO2, and not AGO1, was significantly loaded with miR-133a (Fig. 2B). Taken together, these data indicate that the *off-state* of the Wnt pathway is responsible for the nuclear translocation of AGO2-loaded miR-133a

### AGO2 directly mediates the nuclear shuttling of mature miR-133a

**Figure 3:**
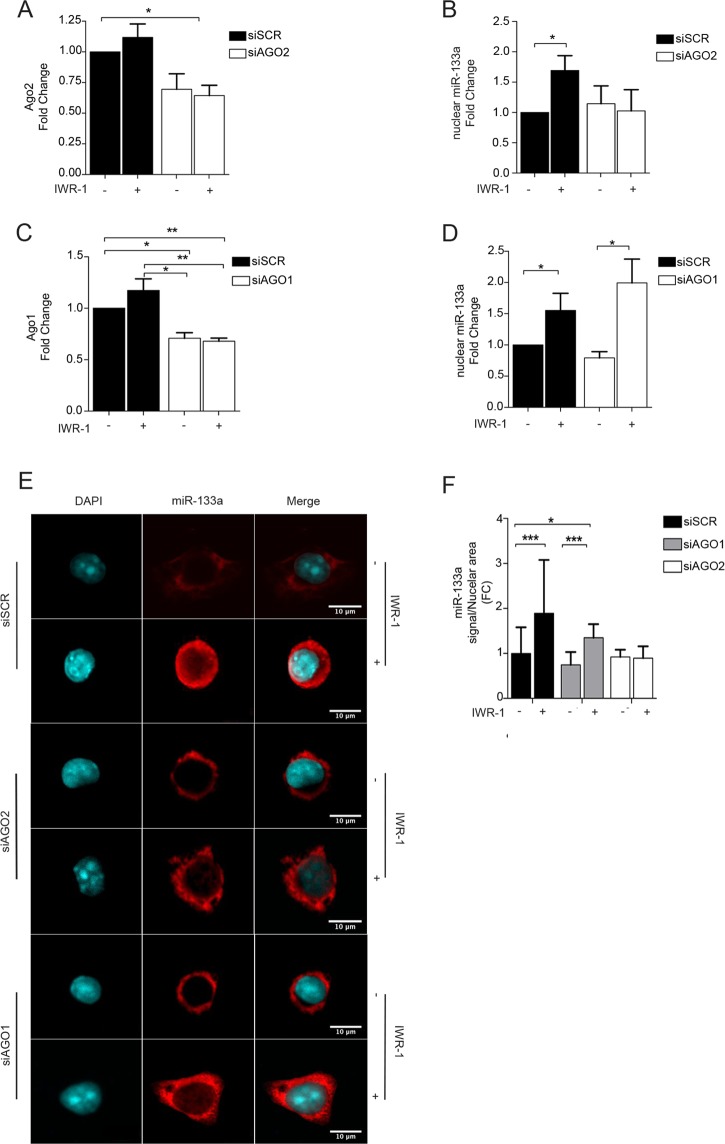
AGO2 is required for the nuclear localization of miR-133a in HL-1 cells in response to IWR-1 treatment. (**A**,**C**) qRT-PCR for *Ago2* and *Ago1* mRNA levels after transient transfection of specific siRNAs. (**B**,**D**) qRT-PCR for mature miR-133a from isolated nuclei of HL-1 cells in presence or absence of IWR-1 (48 h). (**E**) Representative FISH image HL-1 cells transfected with of siRNAs against AGO2, AGO1 and SCR, and treated or not with IWR-1. Red, miR-133a; blue, DAPI. (**F**) Graphic plot of the average miR-133a signal over the nuclear area, in HL-1 cells transfected with siRNAs and treated or not with IWR-1 as indicated. Red, miR-133a; blue, DAPI (n = 6); *p < 0.05; **p < 0.01.

To determine whether AGO2 is crucial for the nuclear translocation of mature miR-133a, the specific effects of siRNAs against AGO2, AGO1, and scramble (SCR) were tested in response to IWR-1 inhibition (Fig. 3A,C). In agreement with the observations above, even though the AGO2 silencing was partial, the nuclear enrichment of miR-133a was significantly compromised in the presence of AGO2 siRNA. On the other hand, no changes were detected in cells depleted for AGO1 (Fig. 3B,D). These results were further confirmed by FISH experiments, showing that transient silencing of AGO2, but not AGO1, significantly affects the nuclear distribution of miR-133a in HL-1 cells (Fig. 3E,F). Overall, these results indicate that AGO2 contributes to the nuclear enrichment of miR-133a in response to the inhibition of the canonical Wnt pathway.

### The karyopherin β family member importin 8 (IPO8) directs the shuttling of the miR-133a/AGO2 complex from the cytosol to the nucleus

**Figure 4:**
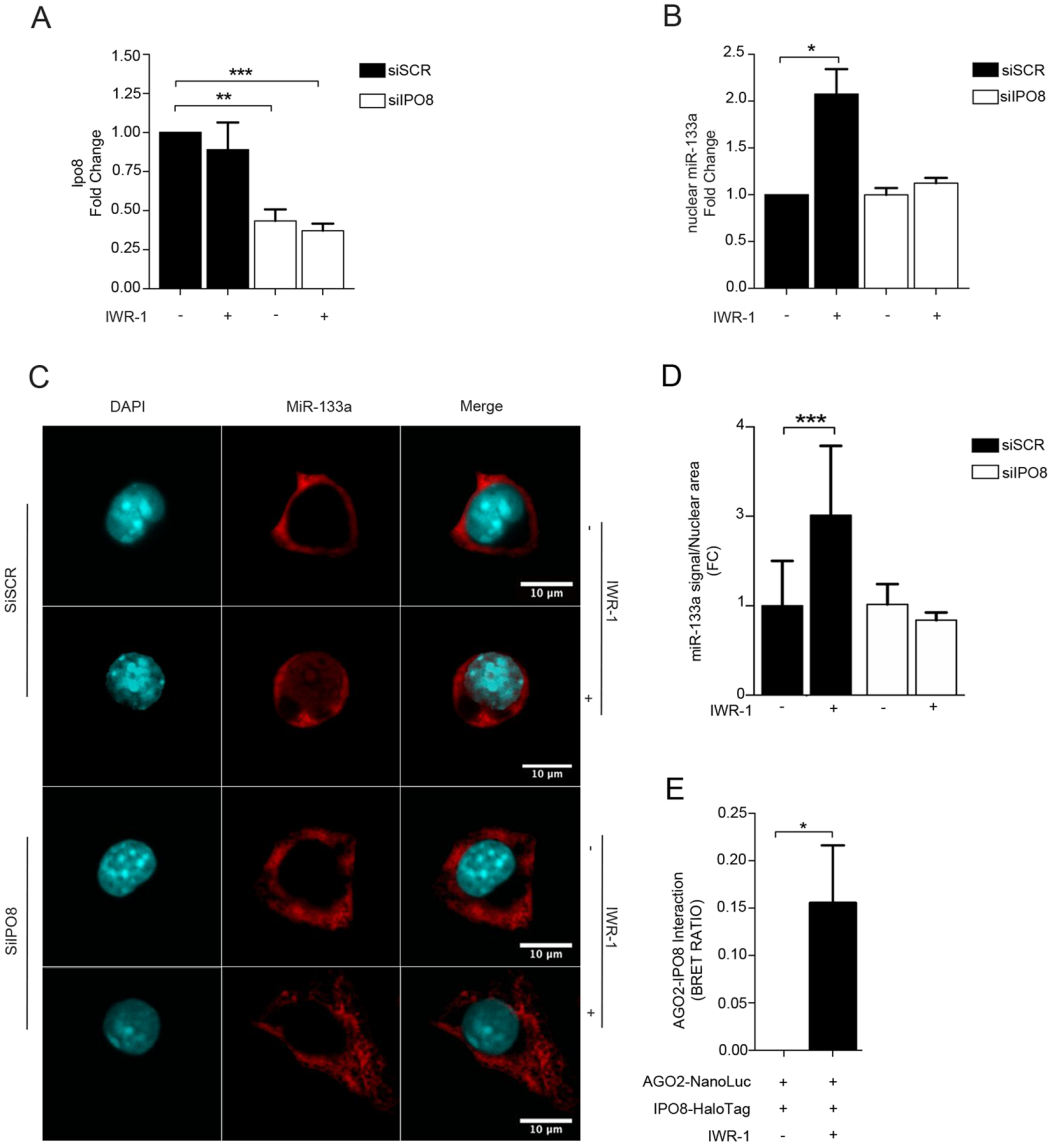
The nuclear enrichment of miR-133a in the Wnt *off-state* is dependent on the interaction between AGO2 and IPO8. (**A**) qRT-PCR for *Ipo8* mRNA after transient transfection of specific siRNAs. (**B**) qRT-PCR for mature miR-133a in isolated nuclei of HL-1 cells treated as indicated. (**C**) Representative FISH image of HL-1 cells transfected with siRNAs and treated or not with IWR-1 as indicated. Red, miR-133a; blue, DAPI (**D**) Graphic plot of average miR-133a/nuclear area in HL-1 cells transfected with siRNAs and treated or not with IWR-1. (**E**) IPO8-AGO2 affinity evaluated in BRET assay in HL-1 cells transfected with AGO2 NanoLuc and IPO8-Halo and treated as indicated (n = 3); *p < 0.05; **p < 0.01; ***p < 0.001.

Having established a link between AGO2 and the nuclear enrichment of miR-133a in the Wnt *off-state*, we were interested in evaluating whether other proteins, beyond the members of miRNA biogenesis pathway, could be involved in this specific cytosol-to-nuclear shuttling. In a recent study, importin 8 (IPO8), a member of the karyopherin β family, was shown to be involved in the transport of mature miRNAs from the cytoplasm to the nucleus in mammalian cells^28^. To determine whether IPO8 also plays a role in the nuclear translocation of mature miR-133a in the Wnt *off-state*, we transiently transfected HL-1 cells with specific siRNAs against IPO8 (Fig. 4A) and determined the nuclear enrichment of mature miR-133a after IWR-1 administration. This revealed that IPO8 depletion reduced the nuclear enrichment of mature miR-133a induced by IWR-1 (Fig. 4B), which was confirmed by FISH (Fig. 4C,D), in which the miR-133a nuclear signal was negatively affected by IPO8 silencing. To further corroborate the involvement of IPO8 in the nuclear shuttling of miR-133a, we performed a bioluminescence resonance energy transfer (BRET) assay to evaluate a potential interaction between AGO2 and IPO8. Notably, we found that an interaction between these two proteins occurs, but only in presence of IWR-1 (Fig. 4E), demonstrating IPO8 as an important component of the machinery responsible for nuclear re-localization of miR-133a in HL-1 cells.

### Nuclear miR-133a is recruited at the Dnmt3b promoter and represses Dnmt3b expression

**Figure 5:**
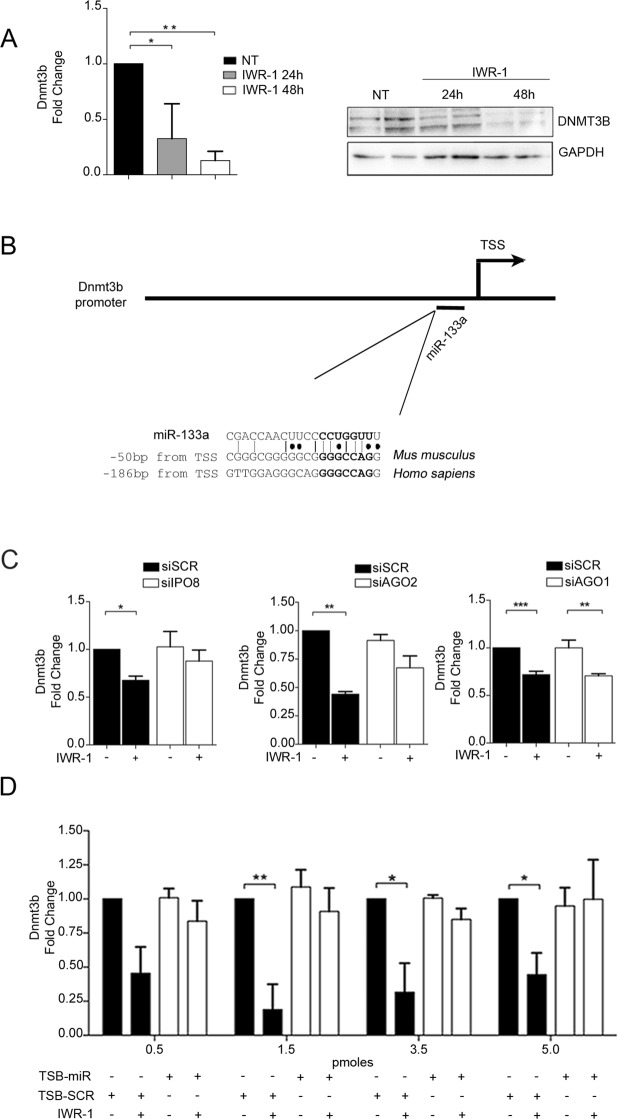
A miR-133a binding site in the *Dnmt3b* promoter is responsible for downregulation of gene transcription. (**A**) qRT-PCR and Western blot analysis (cropped images of the same gel) for DNTM3B in HL-1 cells treated or not with IWR-1. Representative experiments are shown (n = 3). (**B**) Scheme of the *Dnmt3b* promoter and identification of the conserved putative binding site for miR-133a (PBSM). (**C**) qRT-PCR for *Dnmt3b* in HL-1 cells transfected with siRNAs against IPO8, AGO2, or AGO1. (n = 5). (**D**) qRT-PCR for *Dnmt3b* on HL-1 cells transfected with miR-133a target site blocker (TSB-miR) or scramble (TSB-SCR) and treated or not with IWR-1. Representative experiments are shown (n = 4; *p < 0.05; **p < 0.01).

Epigenetic regulation of gene transcription has been shown to play an important role in developmental and differentiation processes as well as in tissue homeostasis^29^, which also applies to the cardiac system where the epigenetic status of key regulatory genes has been shown to be causally linked to the modulation of diverse signalling pathways, including the Wnt cascade^30^. Therefore, we next extended our analyses to understand the epigenetic status of HL-1 cells in the Wnt *off-state*. To address this point, we performed qRT-PCR analyses to determine the expression level of genes encoding major transcription factors and epigenetic enzymes known to be involved in the control of cardiac regulatory genes (Fig. S5). Among the modulated genes, *de novo* DNA methyltransferase 3B (*Dnmt3b*) was the most affected after IWR-1 treatment both at the transcriptional and post-transcriptional level (Fig. 5A). *Dnmt3b* belongs to a family of specific catalytically active enzymes of DNA methyltransferases, which includes “maintenance” methyltransferase (DNMT1) and *de novo* methyltransferases (DNMT3A and DNMT3B). Both types of methyltransferases add methyl groups to CpG residues within gene promoters, thereby modifying the accessibility of DNA to the transcriptional machinery^31^. Accordingly, altered regulation of CpG methylation has been linked to cardiovascular disease development and progression^32,33^.

To understand whether nuclear miR-133a may be directly involved in the negative regulation of *Dnmt3b* expression, we used a bioinformatics approach and scanned the *Dnmt3b* promoter sequence for putative binding sites complementary to miR-133a. Notably, a putative binding site for miR-133a (PBSM) was found at −50 bp from the transcription start site (TSS), which was partially conserved in human (Fig. 5B). To effectively assess whether or not the nuclear enrichment of miR-133a was directly linked to the transcriptional control of *Dnmt3b*, we determined the expression of *Dnmt3b* in the presence of specific siRNAs against AGO2 and IPO8. As expected, abrogation of the AGO2- and IPO8-mediated translocation of miR-133a to the nucleus strongly attenuated the downregulation of *Dnmt3b* (Fig. 5C), while no modulation was detected in the scramble (siSCR) condition. No effect on the expression of *Dnmt3b* was observed in the presence of AGO1 siRNA (Fig. 5C) in agreement with our finding that AGO2 is responsible for the nuclear localization of miR-133a.

To prove that the transcriptional downregulation of *Dnmt3b* is secondary to the binding of miR-133a to the PBSM in the *Dnmt3b* promoter, we transfected HL-1 cells with a specific target site blocker (TSB) oligonucleotide designed to selective anneal to the PBSM (TSB-miR) to compete with miR-133a for binding to the PBSM. TSB-miR prevented the miR-133a downregulation of *Dnmt3b* transcription mediated by administration of IWR-1, while no effects were observed using scramble TSB (TSB-SCR) (Fig. 5D).

Taken together, these results show that miR-133a transcriptionally represses the expression of *Dnmt3b* through direct binding to PBSM within the target gene promoter.

### The miR-133a/AGO2 complex mediates an epigenetic rearrangement within the Dnmt3b promoter and induces DNMT3B to methylate its own promoter via a miR-133a-mediated negative Feed-back loop

Various studies have demonstrated the involvement of AGO-small RNA complexes in the direct regulation of gene transcription^26,34–37^. Kim H. *et al*. demonstrated that the AGO1-loaded miR-704 binds to a targeting site within the *Polr3d* promoter and promotes the tri-methylation of histone H3 lysine 27 (H3K27me3), thereby inducing a miRNA-directed transcriptional gene silencing in mammalian cells^10^. Based on this evidence and our data of a direct binding of miR-133a to the *Dnmt3b* promoter, we further hypothesized that miR-133a in complex with AGO2 can induces a repressive epigenetic status of *Dnmt3b* gene.

**Figure 6:**
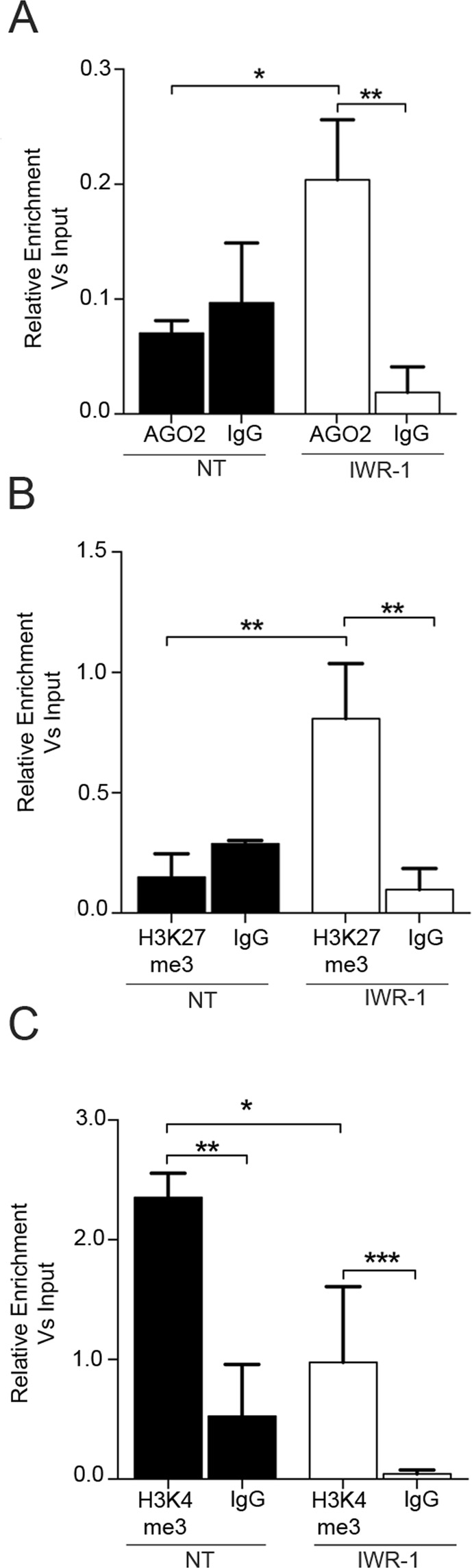
The AGO2/miR-133a complex binds to the *Dnmt3b* promoter and induces epigenetic changes associated with transcriptional repression. (**A**–**C**) ChIP assay evaluating the enrichment of AGO2, H3K27me3, and H3K4me3 in the proximity of PBSM in the *Dnmt3b* promoter. Representative experiments are shown (n = 3); *p < 0.05; **p < 0.01; ***p < 0.001.

To determine whether AGO2 binds to the *Dnmt3b* promoter region, we performed chromatin immunoprecipitation (ChIP) followed by qRT-PCR using primers specific for the PBSM region of the *Dnmt3b* promoter as well as two sets of control primers located outside the PBSM at −1500 bp and +500 bp from the TSS, respectively. As shown in Fig. 6A, IWR-1-dependent enrichment of AGO2 was observed in proximity of the PBSM in the *Dnmt3b* promoter, while no significant changes were observed in the two control regions (Fig. S6). The IWR-1 dependent AGO2 enrichment at the PBSM region was accompanied by a significant increase in the histone repressive marker H3K27me3 (Fig. 6B), while the marker of transcriptional activation H3K4me3, as expected, was more enriched in untreated cells (Fig. 6C). Taken together, our results suggest that miR-133a recruits AGO2 to the PBMS within the *Dnmt3b* promoter, thereby regulating gene transcription through induction of epigenetic changes associated with transcriptional repression.

**Figure 7:**
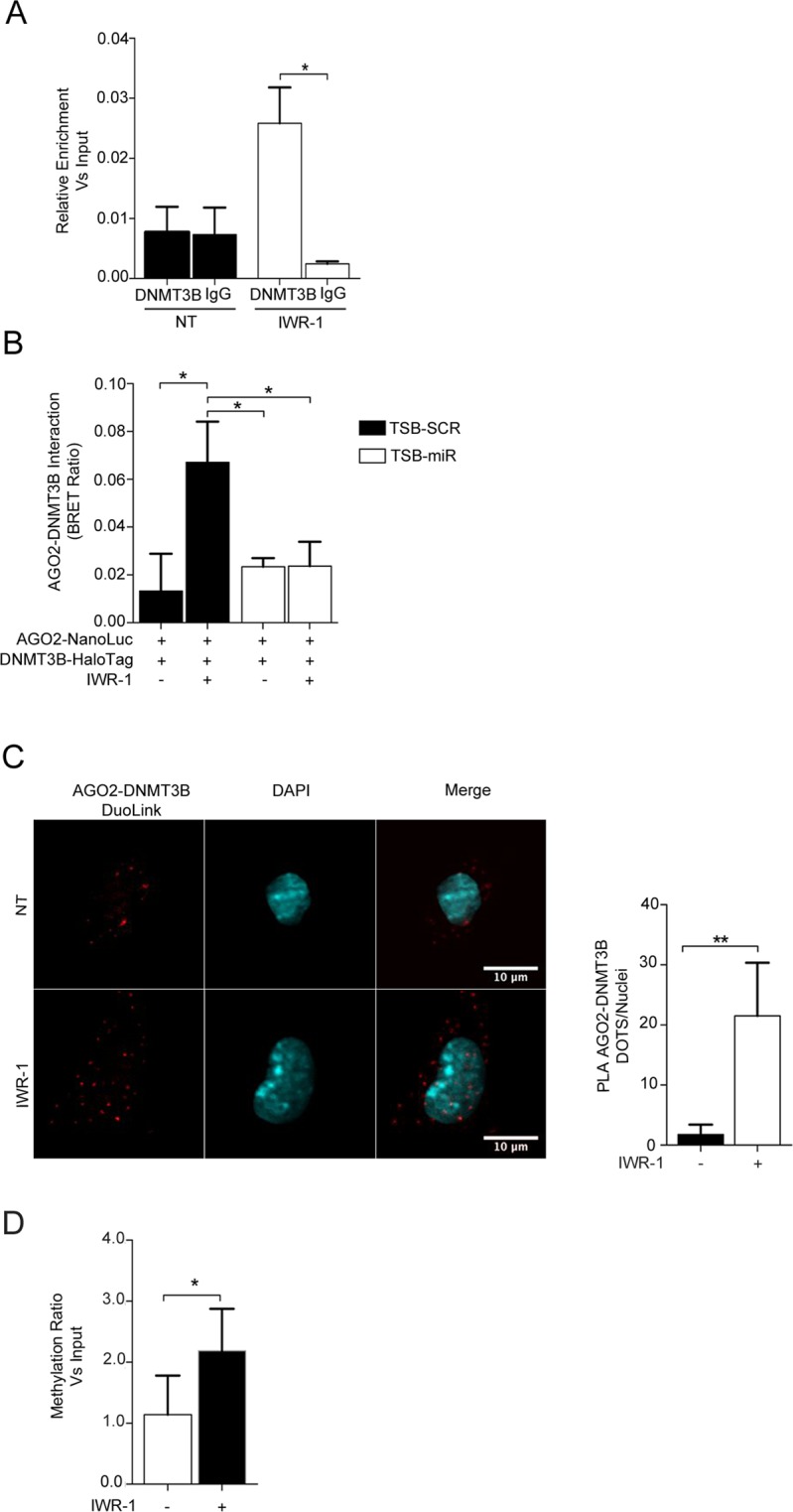
The AGO2/miR-133a complex induces *Dnmt3b* transcriptional gene silencing via a negative feed-back loop dependent on DNMT3B itself. (**A**) ChIP assay for evaluating DNMT3B enrichment in the proximity of PBSM in the *Dnmt3b* promoter. (**B**) DNMT3B-AGO2 protein interaction measured in a BRET assay on HL-1 cells transfected with AGO2 NanoLuc and DNMT3B-Halo and treated as indicated (n = 4). Representative experiments are shown. **p < 0.05. (**C**) Duo-Link assay for the interaction between endogenous AGO2 with DNMT3B (red dots) in HL-1 cells treated or not with IWR-1 and counter-stained with DAPI (blue). Right, graphical plotting of the AGO2/DNMT3B interaction. D) qRT-PCR for detection of DNA methylation in HL-1 cells treated as indicated.

In plants and lower organisms, small interfering RNAs (siRNAs) are known to target specific genomic regions to mediate transcriptional gene silencing^38^. This mechanism relies on the RNA-directed DNA methylation targeting system (RdDM) in which small RNAs of 21–24 nucleotides are incorporated into the AGO4 complex and guide DRM1/2, the homologue of mammalian DNMT3A/B, to its corresponding antisense target within the genomic DNA^39–41^. It remains unclear whether an RdDM is also present in mammals, although there are some studies suggesting that in human cells short hairpin RNAs (shRNAs) can target CpG residues within the promoters of specific genes and promote transcriptional gene silencing through DNA-methyltransferase-dependent mechanisms similar to what observed in plants^42^. Since the miR-133a PBSM in the *Dnmt3b* promoter is located within a CpG-rich region, we assessed whether the induction of the Wnt *off-state* might lead to an enrichment of DNMT3B at the level of the PBMS. In line with our hypothesis, a ChIP assay followed by qRT-PCR revealed a significant enrichment for DNMT3B binding to the PBSM region following IWR-1 administration (Fig. 7A). Furthermore, a BRET assay demonstrated a physical interaction between AGO2 and DNMT3B, which is mediated by the miR-133a binding to PBSM, and administration of TSB-miR completely abolished the AGO2/DNMT3B interaction (Fig. 7B). Physical interaction between endogenous AGO2 and DNMT3B was additionally confirmed by the Duo-Link proximity ligation assay (Fig. 7C). Finally, the methylation ratio of the *Dnmt3b* promoter in proximity to the PBSM was increased after Wnt inhibition with IWR-1 (Fig. 7D).

Taken together, these data suggest that a specific DNMT3B negative-feedback loop is responsible for the miR-133a-mediated silencing of DNMT3B in cardiac cells.

### Nuclear AGO2/miR-133a regulates Dnmt3b transcription during cardiac differentiation of human pluripotent stem cells

**Figure 8:**
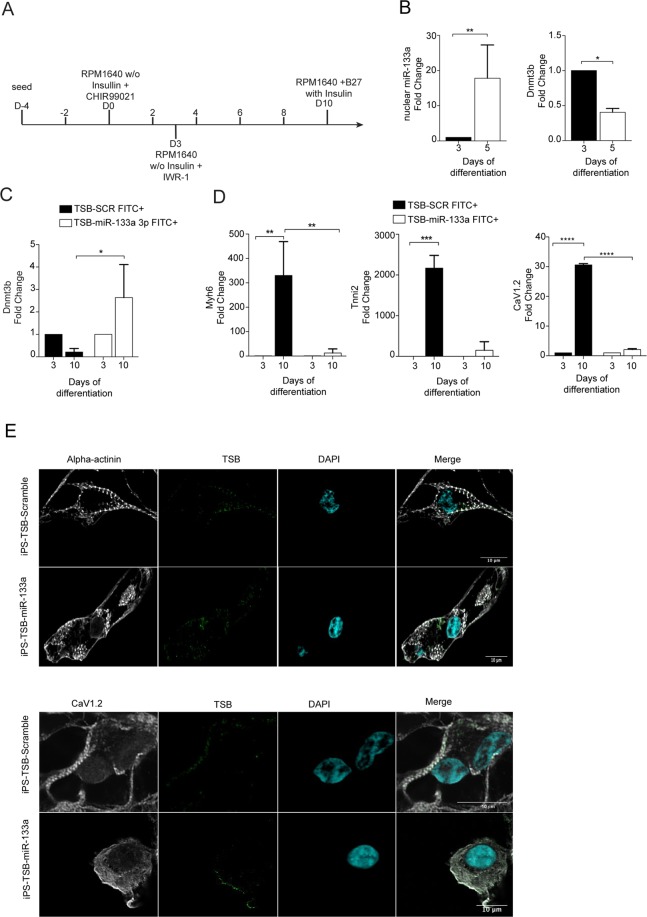
The nuclear re-localization of the AGO2/miR-133a complex is essential for differentiation of hESCs into cardiomyocytes. (**A**) Schematic representation of the cardiac differentiation protocol of hESCs. (**B**) qRT-PCR for miR-133a and *DNMT3B* on isolated nuclei and total cell lysate of differentiating hESCs. (**C**) qRT-PCR for *DNMT3B* on FITC^+^ sorted cells treated as indicated. (**D**) qRT-PCR on *MHY6*, *TNNI2* and *CACNA1C* genes in hESC-CMs (n = 3); *p < 0.05; **p < 0.01; ***p < 0.001. (**E**) Immunostaining for al α-actinin and Ca_v_α1.2 on hESC-CMs at day 15 of cardiac differentiation treated with FITC-conjugated TSB-miR or TSB-SCR.

The Wnt β-catenin signalling pathway is a key regulator of cardiac development^43^ and adult heart remodelling^44^. Additionally, its temporal *on-off* modulation *in vitro* is required, among other stimuli, for the differentiation of pluripotent stem cells (PSCs) towards the cardiac lineage^45,46^. Thus, to provide additional proof of concept in support of the above identified mechanism, our next question was whether miR-133a may relocalizes to the nucleus during the differentiation of PSCs towards the cardiac lineage and whether nuclear miR-133a might play an active role in the transcriptional regulation of *Dnmt3b* transcription also in this model system. To test this hypothesis, we employed RUES2 human embryonic stem cells (hESCs) and verified whether miR-133a was nuclear enriched during cardiac differentiation at the temporal window in which the chemical inhibition of the Wnt canonical signalling (day 3) is normally applied (Fig. 8A). In agreement with the results in HL-1 cells described above, IWR-1 treatment of hESCs induced nuclear enrichment of miR-133a on day 5 of the differentiation protocol (Fig. 8B), which was associated with a strong downregulation of *Dnmt3b* mRNA levels (Fig. 8B).

Methylation of cytosine residues in genomic DNA is critical for regulation of gene silencing during differentiation processes, including those driving early phases of hESC differentiation towards cardiomyocytes (CMs)^47–49^. To support the evidence that miR-133a plays a direct role in controlling *Dnmt3b* transcription and evaluate the potential effect on hESC differentiation, we next applied TSB-miR throughout the differentiation protocol. In particular, FITC-conjugated TSB-miR was administered on day 2 and FITC^+^ cells were sorted 48 hours post IWR-1 treatment (day 3). As shown in Fig. 8C, qRT-PCR from FITC^+^ cells revealed that TSB-miR administration prevented, as expected, the transcriptional repression of the *Dnmt3b* gene compared to TSB-SCR administration. This supports the notion that the regulation of this epigenetic gene in hESCs is directly dependent on miR-133a binding to the PBSM within the *Dnmt3b* promoter.

D*e novo* DNA methylation is known to be important for proper cardiac development, and conditions of abnormal DNA methylation have been shown to lead to the onset of congenital heart disease^50^. To evaluate whether interference with the miR-133a-AGO2-mediated regulation of *Dnmt3b* expression can affect the proper differentiation of hESCs into CMs, we applied FITC-conjugated TSB-miR along the differentiation protocol, as above, and sorted FITC^+^ cells at day 10, which is the temporal window in which CMs start to form. Gene expression analysis for cardiac-specific markers showed that, compared to FITC^+^ cells transfected with TSB-SCR, FITC^+^-TSB-miR transfected cells expressed lower levels of structural cardiac genes involved in cytoskeleton organization and contractility (e.g *Mhy6*; *Tnni2*) as well as calcium signalling (e.g. *cacna1c*) (Fig. 8D). These results were further corroborated by immunofluorescence analyses, showing altered sarcomere and calcium channel organization, as demonstrated by α-actinin and Ca_v_α1 staining, respectively (Fig. 8E). These data show that mature miR-133a relocalizes to the nucleus in the first phases of cardiac induction of hESCs and suggest that modulation of *Dnmt3b* expression by nuclear miR-133a-AGO2 might contribute to proper differentiation and maturation of CMs.

## Discussion

In a plethora of biological contexts, miRNAs act as regulators of transduction cascades, mainly via the well-consolidated post-transcriptional modulation of cytosolic mRNA targets^51^. Recently, additional regulatory mechanisms have been identified supporting the notion that miRNAs can relocalize to the nucleus, where they provide transcriptional control by guiding Argonaute proteins directly to target gene promoters^52^. However, the potential role of miRNAs in the nucleus of cardiac cells is still unclear.

Here we showed that in the Wnt *off-state*, the cardiac enriched miR-133a relocalizes to the nucleus where it directly regulates the transcription of the *Dnmt3b* epigenetic gene. Notably, the repressive control of *Dnmt3b* transcription relies on a self-regulatory circuit loop where, subsequent to miR-133a/Ago2 binding to a specific miR-133a target site within the *Dnmt3b* promoter region, DNMT3B itself represses the methylation of its own promoter.

MiR-133a is clustered with miR-1 and the two miRNAs are co-transcribed as a single primary transcript. However, our work shows that in the Wnt *off-state*, only miR-133a, and not miR-1, shuttles to the nuclear compartment in complex with AGO2. The analysis of two other miRNAs (i.e. miR-19b and miR-34a-5p), which are actively involved within the Wnt signaling, failed to show any clear effect on nuclear relocalization thus supporting the evidence for a miRNA-selective mechanism of cytoplasm-to-nucleus translocation. In line with previous studies reporting an active role of AGOs within the nuclear compartment^36,53,54^, our data revealed that both AGO1 and AGO2, irrespective of the Wnt status, are present in the nucleus of cardiac cells. However, data from transient silencing of AGO1 and AGO2 and RNA-bound nuclear immunoprecipitation experiments showed that only AGO2 is associated with nuclear miR-133a in response to Wnt inhibition. These data indicate AGO2 as a preferential component of the RISC complex, which is functionally required to elicit gene transcription modulation, possibly via stabilization of miRNAs^55^. Nevertheless, identification of the direct regulatory players involved in the selective recruitment and translocation of miR-133a to the nucleus will be the focus of further studies.

Similar to the mechanism of siRNAs directed transcriptional silencing (RdTS) in which promoter targeted siRNAs can transcriptionally silence genes modulation of target gene transcription mediated by nuclear miRNAs is thought to occur via miRNA-DNA interaction within specific regions, such as those present at TSSs^12,37,56,57^. Consistently, we identified a miR-133a complementary site (PBSM) upstream of the transcriptional start site of the *Dnmt3b* gene and demonstrated that the interaction of the nuclear miR-133a-AGO2 complex with PBSM is sufficient for repression of *Dnmt3b* expression. When the interaction was prevented by the application of the competitive TSB-miR, the negative modulation of *Dnmt3b* was abolished. A number of studies have reported that nuclear miRNA bound to promoter regions can regulate target gene expression via recruitment of chromatin-modifying proteins and histone-modifying enzymes to induce epigenetic modifications^12,58^. Our results corroborate this view and support a model in which the miR-133a/AGO2 complex represses the expression of *Dnmt3b* by first inducing an enrichment of the histone repressive mark H3K27me3 and, subsequently, through a negative feedback loop, recruiting DNMT3B to self-methylate its own promoter.

DNA methylation is a well-known epigenetic mechanism, which is essential for the control of stem cell fate. Among the three DNA methyltransferases (DNMT1, DNMT3a, and DNMT3B) important for DNA methylation, DNMT3B is highly expressed in mouse and human ESCs, while its level of expression suddenly decreases as differentiation is induced, allowing for the proper commitment of ESCs towards different lineages^59^. *Dnmt3b* knockout mice die between E13.5 and E16.5 with cardiac ventricular septal defects among others, suggesting that DNMT3B is crucial for cardiac ventricular septum development and function^49,60^. In the present study, we employed a human model of cardiogenesis based on the differentiation of hESCs and confirmed that the transcriptional control of *Dnmt3b* is governed by binding of nuclear miR-133a to the promoter of the gene. Perturbation of this mechanism contributes to an alteration in the differentiation of hESCs towards CMs, as demonstrated by the reduced expression of cardiac-specific genes involved in contractility and calcium handling. Although a more in-depth characterization is required to elucidate the role of nuclear miR-133a during hESC differentiation, which was out of scope of the present study, these data provide a first indication of a mechanistic link between the *off-state* Wnt/β-catenin pathway, the nuclear function of miR-133a, and the *Dnmt3b* gene as a potential checkpoint for cardiac differentiation *in vitro*.

In summary, our study reveals a previously unrecognized role of mature miR-133a, which, upon its relocalization to the nucleus, is directly responsible for epigenetic repression of its target gene, *Dnmt3b*, which is further reinforced by activation of a DNMT3B self-regulatory negative feedback loop. In the light of the increasing interest in miRNA-based therapies, the establishment of more myocardial-directed delivery approaches^61–63^, and the ongoing evaluation of miRNA therapeutics at both preclinical and clinical stages, our findings may provide additional valuable indications for the development of novel miRNA-related strategies targeted to contrast the development of pathological disorders.

## Methods

Detailed procedure are provided in the supplementary information.

### Cell line and culture

HL-1 cells were cultured according to Dr. Claycomb’s instructions. For the modulation of the Wnt canonical pathway, HL-1 cells were stimulated with either 5 μM IWR-1 (Sigma-Aldrich) or 5 μM CHIR99021 (Selleck Chemicals) for 24–48 hours, before being collected.

### Cardiac differentiation of hESCs

RUES2-hESCs were maintained in Essential 8 Medium (Life Technologies) on vitronectin-coated dishes at 37 °C with 5% CO_2,_ as previously described^64^. Cell passaging was carried out by dissociation into single-cell suspension using 0.5 mM EDTA (Gibco Life Technologies). Cardiac induction was achieved using a chemically-defined differentiation protocol as previously described^65^. FACS-Sorting experiments were performed on days 3–10 of the differentiation protocol. All experiments with RUES2-hESCs were carried out in accordance with relevant guidelines and regulations.

### Extraction of nuclear and cytosolic fractions

The nuclear fraction of HL-1 cells was extracted using the PARIS^TM^ kit (Ambion). Cells were washed three times with phosphate-buffered saline (PBS) on ice, followed by centrifugation at 300 × g for 5 min. Cell pellets were resuspended in cell fraction buffer from the PARIS^TM^ kit, incubated on ice for 10 min, and subsequently centrifuged at 500 × g for 5 min at 4 °C. Nuclear pellets were homogenized with the cell disruption buffer from the PARIS^TM^ kit.

### siRNA and target site blocker transfection

siRNA-AGO2 or siRNA-AGO1 pooled with siRNA-IPO8 or siRNA-Scramble (Sigma-Aldrich) were transfected in HL-1 cells using Lipofectamine 2000 (Life Technologies) according to the manufacturer’s instructions and treated with 5 μM IWR-1 (Sigma Aldrich) as described above. Gene expression analysis on total RNA was performed 48 hours after treatment.

A specific “Target site blocker” (TSB) designed for the PBMS in the murine *Dnmt3b* and human *DNMT3B* promoters was obtained from Exiqon. HL-1 cells were transfected with incremental doses of TSB or scramble control using Lipofectamine 2000 (Life Technologies) according to the manufacturer’s instructions and the IWR-1 stimulation with performed as described above. For hESC transfection, TSB or scramble were conjugated with fluorescein (FITC) and transfected on day 2 of cardiac differentiation using ViaFect Reagent (Promega^TM^) according to the manufacturer’s protocol.

### RNA isolation, qRT-PCR, and gene expression analysis

Total or subcellular-derived RNAs were extracted using PureZol Reagent (Bio-Rad). Reverse transcription of RNA for miR-133a-3p (miR-133a in the text), miR-1-1 (miR-1 in the text), miR-34a-5p, miR-19b, miR-206, miR-208a and U6 was performed using the miRCURYLNA™ Universal RT microRNA PCR Polyadenylation and cDNA synthesis kit (Exiqon). Quantitative real time polymerase chain reaction (qRT-PCR) was performed with microRNA LNA™ PCR primers (Exiqon) using the GoTaq Probe qPCR Master Mix (Promega). Relative expression was calculated using the ΔΔ(Ct) method. MiRNA expression in cells or nuclei was normalized to U6 snRNA, while mRNA expression in cells was normalized to GAPDH. Sequences of primers used for qRT-PCR are reported in Supplementary Tables 1 and 2.

### DNA constructs

For the BRET assay, AGO2 and AGO1 cDNAs were cloned into the pNLF1-N vector, while DNMT3B and IPO8 cDNA were cloned into the HaloTag-pFN21A vector. All vectors for Nano-Luciferase and BRET assays were obtained from Promega. All cloning steps were performed using the In-fusion HD Cloning Plus kit (Clontech).

### Western blot analyses

Protein expression was evaluated in total or subfraction lysates by Western blot analysis according to standard procedures. Antibodies against the following proteins were used: AGO2 (clone 11A9) and AGO1 (clone 4B1) both provided by Helmhotzlzentrum Munchen; H3, H3K27me3 (trimethyl K27) and H3K4me3 (trimethyl K4) from Abcam; DNMT3B (52A1018) from Active Motif; and GAPDH (14C10) from Cell Signaling Technology. Image J software (National Institutes of Health) was used for densitometry analysis.

### Nano-BRET assay

The NanoBRET assay was performed as described by the manufacturer (Promega) and previously performed^66^. Briefly, for protein-protein interaction assays, HL-1 transfected cells were treated with 100 nM NanoBRET 618 Ligand (Promega) and signals were detected 6 hours after treatment. Signals were detected using a Synergy H4 instrument (BioTek) and results were analysed using Prism 6.0 software (GraphPad Software, CA).

### Chromatin Imunoprecipitation (ChIP) assay

HL-1 cells were seeded at a density of 1 × 10^6^ on gelatin/fibronectin plates and treated with IWR-1 for 48 hours as previously reported, and processed according to standard procedure. The qRT-PCR was subsequently performed using the primers reported in Supplementary Table 3.

### Evaluation of methylation enrichment

The methylation enrichment (%) of CpG nucleotides was determined using the MethylCollector™Ultra Kit (ActiveMotif), according to the manufacturer’s instruction. The resulting methylated DNA was analysed through qRT-PCR using specific primers to amplify the locus of miR-133a putative binding site.

### AGO2 nuclear pull-down

HL-1 cells were seeded at a density of 1 × 10^6^ on gelatin/fibronectin plates and treated with IWR-1 for 48 hours as previously described. Cells were collected and processed for nuclear extracts using the Nuclear Complex Co-IP Kit, according to the manufacturer’s protocol. The nuclear protein lysate was then incubated with antibodies against AGO2 or IgG, and 20 μl of Dynabeads® Protein G (Thermo Fisher Scientific) overnight at 4 °C with rotation. For protein analysis, samples were subjected to Western blot assays, while for miRNA analysis, the RNA derived from AGO2 or IgG pull-down assay was isolated and subsequently analysed by qRT-PCR.

### Fluorescent ***in situ*** hybridization (FISH) and Duo-Link assay

HL-1 cells were seeded at 5.0 × 10^4^ cells/well on VWR Micro cover slips coated with gelatin/fibronectin in complete Claycomb medium, and incubated overnight at 37 °C with 5% CO_2_. The following day, HL-1 cells were treated with 5 μM IWR-1 for 24–48 hours. MiRCURY LNA™ Detection probe was used to stain for miR-133a or scramble, and tyramide signal amplification (TSA) system (Life technologies) was used to detect miRNA or scramble signal, while nuclei were counterstained with 4′,6-diamidino-2-phenylindole, dihydrochloride (Life Technologies). Fluorescent images were taken with a laser scanning confocal microscope (Olympus, FV1000/SIMS). For he AGO2-DNMT3B interaction, the Duo-Link assay (Sigma Aldrich) was used following the manufacturer’s instructions.

### FACS sorting

FITC positive and negative PSCs-derived cardiomyocytes were sorted using a FACS Aria III instrument (Becton Dickinson, Franklin Lakes, CA, USA). Photomultiplier voltages and laser time delay were checked on a daily basis to ensure the maximum reproducibility of results.

Cell sorting was performed at room temperature using a 100 μm nozzle, 20 psi pressure, 3500 plate voltage and low speed to reduce cell stress to the minimum. Doublet discrimination was performed by plotting events in a FSC-A vs. FSC-H dot plot.

### Statistical analysis

Data are presented as mean ± SD. The normality of data was assessed using the Kolmogorov-Smirnov test. Statistical comparison was performed in at least 3 independent experiments with the Mann-Whitney test, and comparisons between groups were analysed by ANOVA repeated-measures in combination with the Tukey multicomparison. Prism 6.0 software (GraphPad Software) was used to verify the normality of the data and for statistical calculation. A value of P < 0.05 was considered statistically significant.

## Supplementary information

Supplementary Method and data
