# Supplementary material for: Wnt signalling mediates miR-133a nuclear re-localization for the transcriptional control of Dnmt3b in cardiac cells
Source: Sci Rep. 2019 Jun 27;9:9320. doi: 10.1038/s41598-019-45818-4 (PMC6597717; doi:10.1038/s41598-019-45818-4)
Supplement: Supplementary file 1 — Supplementary Method and data [file 41598_2019_45818_MOESM1_ESM.pdf]

Supplementary Material

Wnt signalling mediates miR-133a nuclear re-localization for the transcriptional control of *Dnmt3b* in cardiac cells

Vittoria Di Mauro[1, 2, 3], Silvia Crasto[2,3], Federico Simone Colombo[3], Elisa Di Pasquale[2, 3] and Daniele Catalucci[2, 3,*]

[1]University of Milan Bicocca, Piazza dell'Ateneo Nuovo 1, 20126 Milan, Italy

[2]CNR-IRGB UOS Milan, Via Fantoli 15/16, 20138 Milan, Italy

[3]Humanitas Clinical and Research Center, via Alessandro Manzoni 113, 20089 Rozzano, Milan, Italy

*Correspondence: daniele.catalucci@cnr.it

Cardiac differentiation of hESCs

RUES2-hESCs were maintained in Essential 8 Medium (Life Technologies) on vitronectin-coated dishes at 37°C with 5% CO_2, as previously described [58]. Cell passaging was carried out by dissociation into single cell suspension using 0.5 mM EDTA (Gibco Life Technologies).
Cardiac induction was achieved using a chemically-defined differentiation protocol as previously described [59]. Briefly, cells were plated onto Growth Factor Reduced (GFR) Matrigel-coated plates and cultured to 90-100% cell confluence. Cells were then treated for 24 hours with 12 µM CHIR99021 (Selleck Chemicals) in RPMI+B27 supplement without insulin, which activates the Wnt signalling pathway and initiates cardiac commitment. The next day, media was replaced with RPMI+B27 without insulin. Treatment with 5 µM IWR-1 (Sigma-Aldrich) was performed on day 3 for 48 hours. Cells were then maintained in RPMI+B27 without insulin until day 10 when media was replaced with RPMI+B27 with insulin. FACS-Sorting experiments were performed on days 3-10 of the differentiation protocol. All experiments with RUES2-hESCs were carried out in accordance with relevant guidelines and regulations.

Extraction of nuclear and cytosolic fractions

The nuclear fraction of HL-1 cells was extracted using the PARIS™ kit (Ambion). Cells were washed three times with phosphate-buffered saline (PBS) on ice, followed by centrifugation at 300 X g for 5 min. Cell pellets were resuspended in cell fraction buffer from the PARIS™ kit, incubated on ice for 10 min, and subsequently centrifuged at 500 X g for 5 min at 4°C. Nuclear pellets were homogenized with the cell disruption buffer from the PARIS™ kit.

siRNA and Target site blocker transfection

siRNA-AGO2 or siRNA-AGO1 pooled with siRNA-IPO8 or siRNA-Scramble (Sigma-Aldrich) were transfected in HL-1 cells using Lipofectamine 2000 (Life Technologies) according to the manufacturer's instructions. 6 hours after transfection, culture medium was changed and HL-1 cells were treated with 5 µM IWR-1 (Sigma Aldrich) as described above. Gene expression analysis on total RNA was performed 48 hours after treatment, A specific "Target site blocker" (TSB) designed for the PBMS in the murine Dnmt3b and human DNMT3B promoters was obtained from Exiqon. HL-1 cells were transfected with incremental doses of TSB or scramble control using Lipofectamine 2000 (Life Technologies) according to the manufacturer's instructions. 6 hours after transfection, the medium was changed and the IWR-1 stimulation with performed as described above. For hESC transfection, TSB or scramble were conjugated with fluorescein (FITC) and transfected on day 2 of cardiac differentiation using ViaFect Reagent (Promega™) according to the manufacturer's protocol.

RNA isolation, qRT-PCR, and gene expression analysis

Total or subcellular-derived RNAs were extracted using PureZol Reagent (Bio-Rad). Reverse transcription of RNA for miR-133a-3p, miR-1-1, miR-34a-5p, miR-19b and U6 was performed using the miRCURYLNA™

Universal RT microRNA PCR Polyadenylation and cDNA synthesis kit (Exiqon).
Quantitative real time polymerase chain reaction (qRT-PCR) was performed with microRNA LNA™ PCR primers (Exiqon) using the GoTaq Probe qPCR Master Mix (Promega). Relative expression was calculated using the ΔΔ(Ct) method. MiRNA expression in cells or nuclei was normalized to U6 snRNA, while mRNA expression in cells was normalized to GAPDH. Sequences of primers used for qRT-PCR are reported in Supplementary Table 1 and Supplementary Table 2.

DNA constructs
For the BRET assay, Ago2 and Ago1 cDNAs were cloned into the pNLF1-N vector, while DNMT3B and IPO8 cDNA were cloned into the HaloTag-pFN21A vector. All vectors for Nano-Luciferase and BRET assays were obtained from Promega. All cloning steps were performed using the In-fusion HD Cloning Plus kit (Clontech).

Western blot analyses
Protein expression was evaluated in total or subfraction lysates by Western blot analysis according to standard procedures. Samples obtained from treated or non-treated cells were homogenized in RIPA buffer (150 mM NaCl, 10 mM Tris-HCl pH 7.2, 0.1% SDS, 1% Triton-X100, 5 mM EDTA, 100 μM Na_3VO_4, 10 mM NaF, and 1X Protease inhibitor (Thermo Fisher Scientific)), loaded onto 8- or 12% Tris-Glycine Gels, separated by electrophoresis, and transferred to a nitrocellulose membrane (Bio-Rad). Antibodies against the following proteins were used: Ago2 (clone 11A9) and Ago1 (clone 4B1) both provided by Helmhotzlzentrum Munchen; H3, H3K27me3 (trimethyl K27) and H3K4me3 (trimethyl K4) from Abcam; DNMT3B (52A1018) from Active Motif; and GAPDH (14C10) from Cell Signaling Technology. Image J software (National Institutes of Health) was used for densitometry analysis. Antibody concentrations used were 2 μg/μl for AGO2 and AGO1; 1 μg/μl for DNMT3B; H3K27me3; H3K4me3 and 0.1 μg/μl for GAPDH
and H3.

Nano-BRET assay
The NanoBRET assay was performed as described by the manufacturer (Promega) [60]. Briefly, for proteinprotein interaction assays, HL-1 transfected cells were treated with 100 nM NanoBRET 618 Ligand (Promega) and signals were detected 6 hours after treatment. Signals were detected using a Synergy H4 instrument (BioTek) and results were analysed using Prism 6.0 software (GraphPad Software, CA).

Chromatin Imunoprecipitation (ChIP) assay
For chromatin immunoprecipitation, HL-1 cells were seeded at a density of 1 x 10^6 on gelatin/fibronectin plates and treated with IWR-1 for 48 hours as previously reported. On the day of harvest, cells were crosslinked with 1% of formaldehyde for 10 min. The cross-linking reaction was stopped with 0.125 M Glycine for 5 min after which cells were washed with cold PBS and collected into IP Buffer (2 volumes of SDS Buffer (100 mM NaCl, 50 mM Tris-HCl pH 8.1, 5 mM EDTA pH 8.0, 0.5% SDS), and 1 volume of Triton Dilution Buffer (100 mM NaCl, 100 mM Tris-HCl pH 8.1, 5 mM EDTA, 5% Triton X-100)). Samples were sonicated using a Bioruptor® Plus sonication device, clarified by centrifugation, and subsequently checked on a 0.8% agarose gel following DNA purification. 20% of clarified lysates was saved as input, before addition of 20 μl of Dynabeads® Protein G (Thermo Fisher Scientific) and specific antibodies (AGO2; DNMT3B; H3K4me3; H3K27me3) and incubation overnight at 4°C. The day after, samples were washed 3 times with Mixed Micelle Buffer (20 mM Tris-HCl pH 8.0, 150 mM NaCl, 5 mM EDTA pH 8.0, 1% Triton X-100, 0.2% SDS), 2 times with Buffer 500 (50 mM HEPES, 0.1% Deoxycholic Acid, 1% Triton X-100, 500 mM NaCl, 1 mM EDTA pH 8.0); and 1 time with LiCl detergent buffer (10 mM Tris-HCl pH 8.0; 0.5% Deoxycholic Acid, 0.5% NP40, 250 mM LiCl). Subsequently, elution was performed in elution buffer (1% SDS and 100 mM $NaHCO_3$) after which de-cross-linking was performed by addition of 200 mM NaCl and incubation at 65°C overnight. The following day, samples were incubated with 20 ug/ml of Proteinase K at 45°C for 2 hours, where after DNA was extracted by ethanol precipitation following treatment with UltraPure™ Phenol:Chloroform:Isoamyl Alcohol (25:24:1, v/v) (Thermo Fisher Scientific). qRT-PCR was subsequently performed using the primers reported in Supplementary Table 3.
Evaluation of methylation enrichment The methylation enrichment (%) of CpG nucleotides was determined using the MethylCollector™Ultra Kit (ActiveMotif), according to the manufacturer's instruction. The resulting

methylated DNA was analysed through qRT-PCR using specific primers to amplify the locus of miR-133a putative binding site.

AGO2 nuclear pull-down

HL-1 cells were seeded at a density of 1 x 10^6 on gelatin/fibronectin plates and treated with IWR-1 for 48 hours as previously described. Cells were collected and processed for nuclear extracts using the Nuclear Complex Co-IP Kit, according to the manufacturer's protocol. The nuclear protein lysate was then incubated with antibodies against AGO2 or IgG, and 20 µl of Dynabeads® Protein G (Thermo Fisher Scientific) overnight at 4 °C with rotation. For protein analysis, samples were subjected to Western blot assays, while for miRNA analysis, the RNA derived from AGO2 or IgG pull-down assay was isolated and subsequently analysed by qRT-PCR.

Fluorescent in situ hybridization (FISH) and Duo-Link assay

HL-1 cells were seeded at 5.0 X 10^4 cells/well on VWR Micro cover slips coated with gelatin/fibronectin in complete Claycomb medium, and incubated overnight at 37°C with 5% CO_2. The following day, HL-1 cells were treated with 5 µM IWR-1 for 24-48 hours. Cultured cells were fixed for 5 min in 4% paraformaldehyde at room temperature, after which cells were permeabilized with PBS containing 0.2% Triton X-100 for 5 min. MiRCURY LNA™ Detection probe was used to stain for miR-133a-3p or scramble, and tyramide signal amplification (TSA) system (Life technologies) was used to detect miRNA or scramble signal. After three washes with PBS, nuclei were counterstained with 4', 6-diamidino-2-phenylindole, dihydrochloride (Life Technologies) for 5 min at room temperature. Fluorescent images were taken with a laser scanning confocal microscope (Olympus, FV1000/SIMS). The AGO2-DNMT3B interaction, the Duo-Link assay (Sigma Aldrich) was used following the manufacturer's instructions.

FACS Sorting

FITC positive and negative PSCs-derived cardiomyocytes were sorted using a FACS Aria III instrument (Becton Dickinson, Franklin Lakes, CA, USA). Photomultiplier voltages and laser time delay were checked on a daily basis to ensure the maximum reproducibility of results.

Cell sorting was performed at room temperature using a 100 µm nozzle, 20 psi pressure, 3500 plate voltage and low speed to reduce cell stress to the minimum. Doublet discrimination was performed by plotting events in a FSC-A vs. FSC-H dot plot.

Statistical Analysis

Data are presented as mean ± SD. The normality of data was assessed using the Kolmogorov-Smirnov test. Statistical comparison was performed in at least 3 independent experiments with the Mann-Whitney test, and comparisons between groups were analysed by ANOVA repeated-measures in combination with the Tukey multicomparison. Prism 6.0 software (GraphPad Software) was used to verify the normality of the data and for statistical calculation. A value of $P < 0.05$ was considered statistically significant.

Supplementary Figures

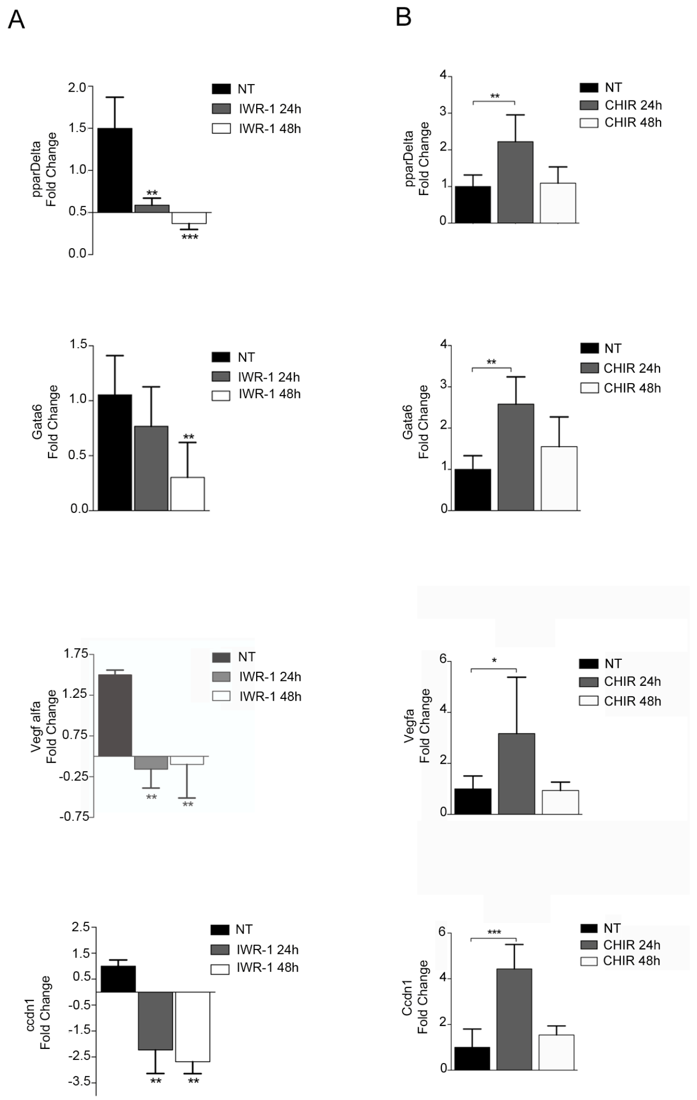

Supplementary Figure 1. Analysis of canonical Wnt/β-catenin signalling pathway target genes in HL-1 cells following treatment with IWR-1 or CHIR99021. **A)** and **B)** qRT-PCR for mRNA levels of Pparδ, Gata6, Vegfα and Ccdn1 in total lysate of HL-1 cells treated as indicated. (n=3); *$p < 0.05$; **$p < 0.01$; ***$p < 0.001$.

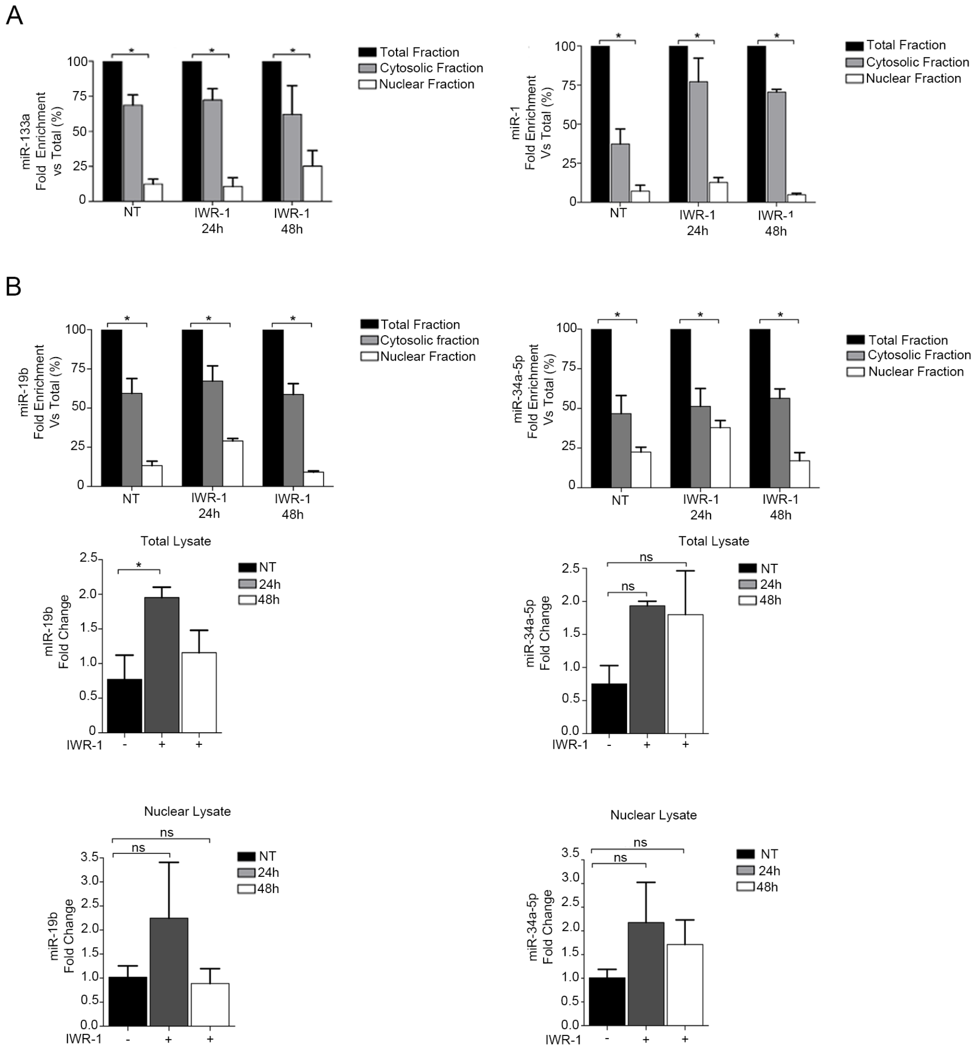

Supplementary Figure 2. Analysis of profile distribution of miR-133a, miR-1, miR-19b and miR-34a-5p following treatment with IWR-1. A) qRT-PCR for mRNA levels of miR-133a-3p and miR-1 in cellular sub-fraction lysates expressed as fold enrichment (%) vs total cell mRNA (10% of lysate). **B)** qRT-PCR for mRNAlevels of miR-19b and miR-34a-5p in cellular sub-fraction lysates expressed as fold enrichment (%) vs total cell mRNA, and in total and nuclear cellular lysates (n=3); *$p < 0.05$

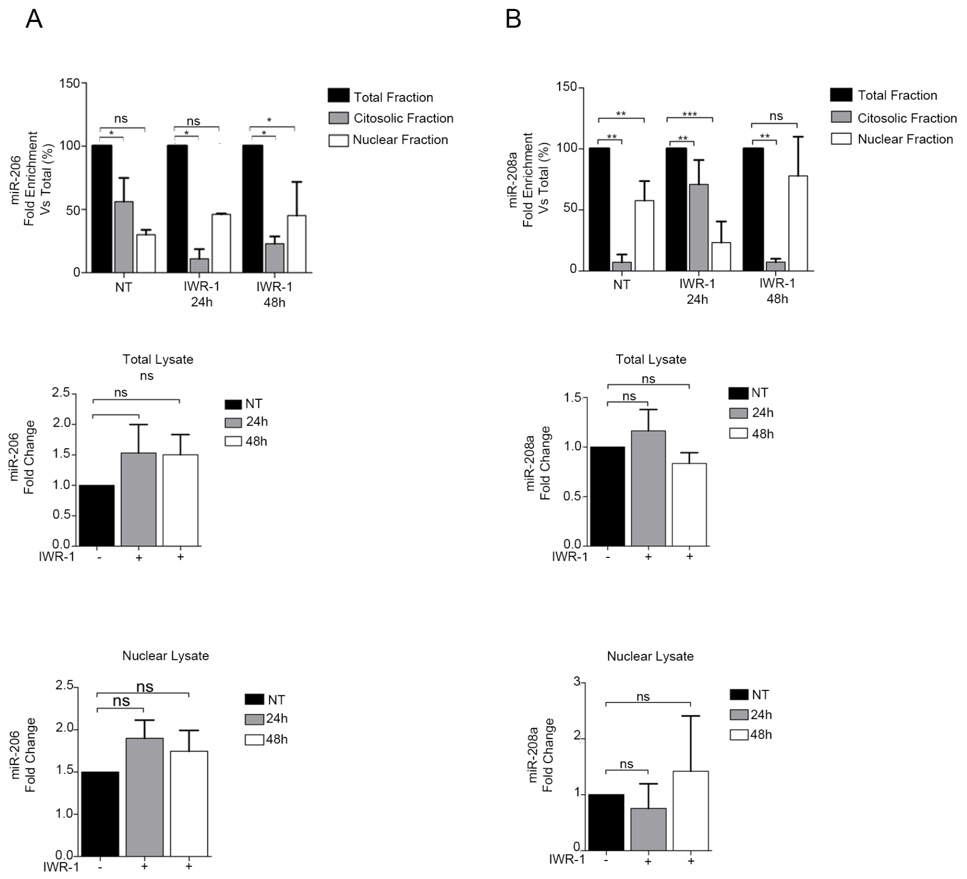

Supplementary Figure 3. Analysis of profile distribution of miR-206 and miR-208a following treatment with IWR-1. A) qRT-PCR for mRNA levels of miR-206 in cellular sub-fraction lysates expressed as fold enrichment (%) vs total cell mRNA (10% of lysate) and in total and nuclear cellular lysates. **B)** qRT-PCR for mRNA levels of miR-208a in cellular sub-fraction lysates expressed as fold enrichment (%) vs total cell mRNA and in total and nuclear cellular lysates. (n=3); $*p < 0.05$; $**p < 0.01$; $***p < 0.005$

.

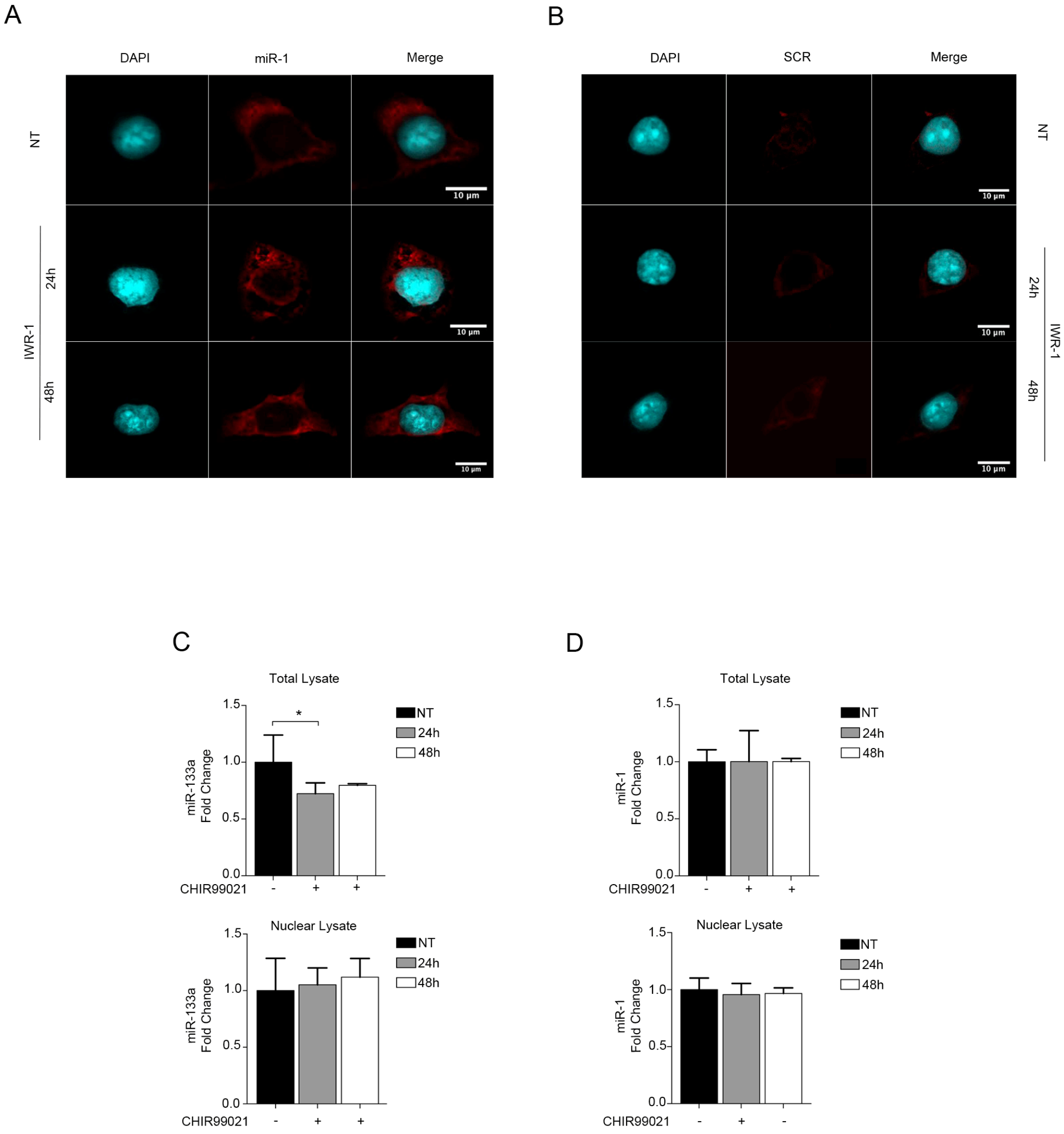

Supplementary Figure 4. Sub-cellular distribution of mature miRNAs after chemical modulation of the Wnt/β-catenin signalling pathway in HL-1 cells. A) and **B)** Representative FISH images of HL-1 cells treated or not with IWR-1, stained with LNA probe for miR-1 or LNA-Scramble probe (red) and DAPI (blue). **C)** and **D)** qRT-PCR for mRNA levels of miR-133a-3p and miR-1 in total cell lysate and the purified nuclear fraction. (n=5); $*p < 0.05$.

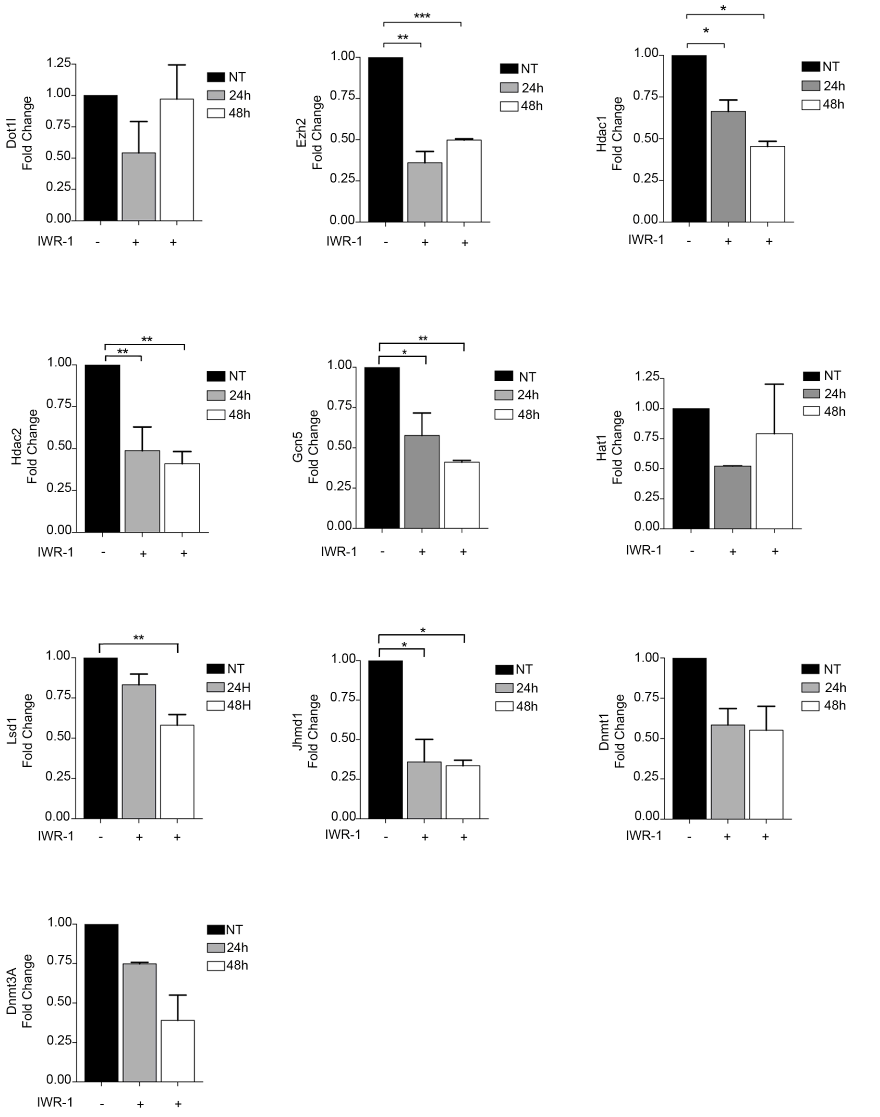

Supplementary Figure 5. Gene expression analysis for the epigenetic enzymes following inactivation of the canonical Wnt pathway. qRT-PCR on HL-1 total cell lysate treated or not with IWR-1. (n=3); $*p < 0.05$; $**p < 0.01$; $***p < 0.001$.

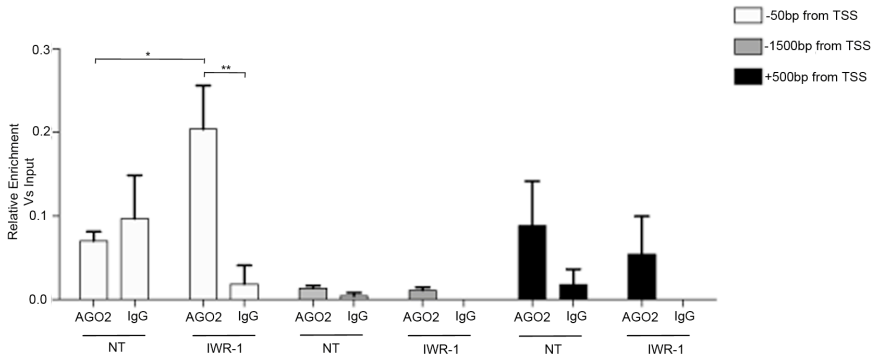

Supplementary Figure 6. Association of AGO2/miR-133a-3p with the Dnmt3b gene following inactivation of the canonical Wnt pathway in HL-1 cells. ChIP assay evaluating the enrichment of AGO2 in proximity of the miR-133a-3p PBSM in the Dnmt3b promoter -50bp, at -1500bp and +500bp from the TSS. Representative experiments are shown (n=3); *$p < 0.05$; **$p < 0.01$.

Supplementary Tables

Supplementary Table 1. Sequence primers for murine genes

Gene	Species	Sequence
Dnmt3b	*Mus musculus*	ACTTGGTGATTGGTGGAAGC<br>CCAGAAGAATGGACGGTTGT
Dnmt3a	*Mus musculus*	ACTTGGAGAAGCGGAGTGAA<br>CTGTTCTTTGCCCTCTCCTG
Dnmt1	*Mus musculus*	CCACCACCAAGCTGGTCTAT<br>TGCCACCAAACTTCACCATA
Dnmt3l	*Mus musculus*	GTTCCTGGAGTCCCTCTTCC<br>CCATGGCATTGATCCTCTCT
Ep300	*Mus musculus*	TGATCCTGACCCTCTCATCC<br>GCACTCGTTGCAGGTGTAGA
Hdac5	*Mus musculus*	AGTGAGAGCACCCAGGAAGA<br>GTACACCTGGAGGGGCTGTA
Hdac1	*Mus musculus*	TTGTCAGGGTCCTCCTCATC<br>TCCAACATGACCAACCAGAA
Hdac2	*Mus musculus*	TGGAGGAGGCTACACAATCC<br>TTTGAACACCAGGTGCATGT
Ezh2	*Mus musculus*	AGACGTCCAGCTCCTCTGAA<br>ATCCTCAGTGGGAACAGGTG
Dot1l	*Mus musculus*	GGTTGTCACCTGCCAAGATT<br>TGAGCTTCATGCTGTTGGAC
Lsd1	*Mus musculus*	CGAATGACCTCTCAGGAAGC<br>GCTGGAGAGTGGCTTCAAAC
Jhdm1	*Mus musculus*	CTACCTCAGCCGCAAAGAAC<br>CTGAGGTCGAGGCTTACTGG
Hat1	*Mus musculus*	AGAGTGCCGTGGAGAAGAAA<br>TTTCATCATCCCCAAAGAGC
Gcn5	*Mus musculus*	ATTCGAGAGACAGGCTGGAA<br>ATGGGGAAACGGATAACCTC
Ipo8	*Mus musculus*	CGTGACAACATTGTGGAAGG<br>GTAAACTGCCAAGCCAGCTC
Gapdh	*Mus Musculus*	ACCCAGAAGACTGTGGATGG<br>CACATTGGGGGTAGGAACAC
U6	*Mus musculus*	TTCACGAATTTGCGTGTCAT<br>CGCTTCGGCAGCACATATAC
Ago2	*Mus musculus*	AAGTCGGACAGGAGCAGAAA<br>GAAACTTGCACTTCGCATCA
Ago1	*Mus musculus*	TCGGAAGATTTCCAAGGATG<br>GTTGCCATTCCCAAGAGTGT

Supplementary Table 2. Sequence Primers for Human genes

Gene	Species	Sequence
**DNMT3B**	Homo sapiens	TTGAATATGAAGCCCCCAAG<br>GGTTCCAACAGCAATGGACT
**GAPDH**	Homo sapiens	CGACCACTTTGTCAAGCTCA<br>AGGGGTCTACATGGCAACTG
**TNNI2**	Homo sapiens	AGTGGGAAGAGGCAGACTGA<br>CGAACTTCTCTGCCTCCAAG
**TNNI3**	Homo sapiens	GATCTCTGCAGATGCCATGA<br>CAGTAGGCAGGAAGGCTCAG
**CACNA1C**	Homo sapiens	TTCACATTTCTTCCTCTTCGTG<br>CGTGGGCTCCCATAGTTG
**MHY7**	Homo sapiens	CTTCAACCACCACATGTTCG<br>GGCTTCTGGAAATTGTTGGA
**MHY6**	Homo sapiens	TGTGTCACCGTCAACCCTTA<br>TGGCTGCAATAACAGCAAAG

Supplementary Table 3. Sequence primers for Chromatin Immunoprecipitation Assay (ChiP)

Genome position	Species	Sequence primer
**chr2:153649260+153649624**	*Mus musculus*	AAGGCATAGGGGGCCCAGG<br>TTGGGCCGCTTAACCCCG
**chr2:153647976+153648322**	*Mus musculus*	AGGCCCAACTCTTACCCCTA<br>GTGCCTTGGGCTGTGATAAT
**chr2:153662009+153662128**	*Mus musculus*	CAATGAAGGGAGACAGCAGA<br>CTCTGGTCTCTGGTGTGCAG
